# Airway CD8^+^CD161^++^TCRvα7.2^+^ T Cell Depletion During Untreated HIV Infection Targets CD103 Expressing Cells

**DOI:** 10.3389/fimmu.2019.02003

**Published:** 2019-08-21

**Authors:** Leonard Mvaya, Andrew Mwale, Annemarie Hummel, Joseph Phiri, Raphael Kamng'ona, David Mzinza, Elizabeth Chimbayo, Rose Malamba, Anstead Kankwatira, Henry C. Mwandumba, Kondwani C. Jambo

**Affiliations:** ^1^Malawi-Liverpool-Wellcome Trust Clinical Research Programme, University of Malawi College of Medicine, Blantyre, Malawi; ^2^Department of Clinical Sciences, Liverpool School of Tropical Medicine, Liverpool, United Kingdom

**Keywords:** airway, HIV, CD103, CD8 T cell, adult

## Abstract

HIV-infected adults are at an increased risk to lower respiratory tract infections (LRTIs). CD8^+^CD161^++^TCRvα7.2^+^ T cells are an innate-like T cell subset that are thought to play an important role in early defense against pathogens in the respiratory tract. HIV infection leads to irreversible depletion of these cells in peripheral blood, however, its impact on this subset in the human airway is still unclear. Here, we show presence of CD103 expressing CD8^+^CD161^++^TCRvα7.2^+^ T cells in the airway that exhibited a distinct cytokine functional profile compared to their CD103^−^ airway counterparts and those from peripheral blood. These CD103 expressing airway CD8^+^CD161^++^TCRvα7.2^+^ T cells were selectively depleted in untreated HIV-infected adults compared to healthy controls. Their frequency was positively correlated with frequency of airway CD4^+^ T cells. Furthermore, the frequency of airway CD8^+^CD161^++^TCRvα7.2^+^ T cells was also inversely correlated with HIV plasma viral load, while suppressive antiretroviral therapy (ART) resulted in restoration of airway CD8^+^CD161^++^TCRvα7.2^+^ T cells. Our findings show that CD103 expressing airway CD8^+^CD161^++^TCRvα7.2^+^ T cells are functionally distinct and are preferentially depleted during untreated asymptomatic HIV infection. Depletion of CD103 expressing airway CD8^+^CD161^++^TCRvα7.2^+^ T cells, at a major portal of pathogen entry, could partly contribute to the increased propensity for opportunistic LRTIs observed in untreated HIV-infected adults.

## Introduction

HIV-infected individuals are at an increased risk to lower respiratory tract infections (LRTIs) (1, 2), which account for 75–98% of lung complications in untreated HIV-infected adults worldwide (3, 4). This susceptibility to LRTIs is largely attributed to HIV-induced disruption of lung immunity, including global alteration in airway immune cell homeostasis (5), reduced frequency of respiratory antigen-specific airway CD4^+^ T cells (6, 7), as well as, impaired alveolar macrophage function (6, 8). While these immune cell perturbations partly underlie propensity for LRTIs in HIV-infected individuals, the impact of HIV infection on other important cells involved in early defense (9, 10), such as airway CD161^++^TCRvalpha (α)7.2^+^ T cells, is not well defined.

CD161^++^TCRvα7.2^+^ are classical markers for Mucosal-Associated Invariant T (MAIT) cells, which are innate-like T cells present in the liver, blood and mucosal tissues including gut, female genital tract (FGT) and the lung (11–14). CD161^++^TCRvα7.2^+^ T cells have characteristics of innate cells and a degree of sophistication possessed by adaptive lymphocytes. They express a semi-invariant T cell receptor, which recognizes microbial vitamin B2 (riboflavin) metabolites (5-(2-oxoethylideneamino)-6-D-ribitylaminouracil or 5-OP-RU), presented via major histocompatibility complex (MHC) class I-related (MR) 1 molecule (14–17). CD161^++^TCRvα7.2^+^ T cells are present in three main subsets, CD4^−^CD8^−^ (DN), CD4^+^, and CD8^+^ phenotypes (18, 19). The majority of the CD161^++^TCRvα7.2^+^ T cells in peripheral blood are actually of the CD8^+^ phenotype (18). Mature CD161^++^TCRvα7.2^+^ T cells display an effector memory phenotype (20), are pre-armed with pro-inflammatory and cytolytic effector molecules (12, 21). This allows them to either lyse infected cells or activate phagocytes very early after infection (22, 23). Furthermore, CD161^++^TCRvα7.2^+^ T cells contribute to regulation of mucosal barrier integrity by secreting IL-22, which promotes epithelial cell proliferation and epithelial tight junction protein expression (24, 25). These qualities highlight the importance of CD161^++^TCRvα7.2^+^ T cells in antimicrobial defense and preservation of mucosal barrier integrity.

CD161^++^TCRvα7.2^+^ T cells in the human lower respiratory tract (LRT) are thought to play an important role in defense against respiratory pathogens. *Streptococcus pneumoniae* and *Mycobacterium tuberculosis* both induce CD161^++^TCRvα^+^ T cell responses through MR1-dependent pathways (16, 26). In patients with active pulmonary TB, CD161^++^TCRvα7.2^+^ T cells are enriched in the lung (16) and decreased in blood (16, 27, 28). It has been shown that decrease in MAIT cells frequencies is linked to expression of PD-1 on MAIT cells during HIV and chronic hepatitis C virus (HCV) infection (29, 30). It was suggested that this expression of PD-1 potentially induces inhibition of MAIT cell proliferation and function due to immune exhaustion (31). In an experimental murine *M. tuberculosis* infection, mice over-expressing CD161^++^TCRvα7.2^+^ T cells have lower bacilli load compared to MR1 knockout (KO) mice (32). This effect of CD161^++^TCRvα7.2^+^ T cells in the lung happens early in infection. In a *M. bovis* pulmonary infection model, higher bacterial burdens are only observed at day 10 in MR1 KO mice compared to wild type mice (33), but not at day 30, suggesting that the impact of CD161^++^TCRvα7.2^+^ T cells in controlling bacterial load is much more significant in early than later stages of infection. An intranasal infection of *Francisella tularensis* live-vaccine strain (LVS) in wild-type and MR1 KO mice, has also established that CD161^++^TCRvα7.2^+^ T cells have a direct early antibacterial effect in the lung and a sustained impact on development of effective adaptive mucosal immune response (10). Taken together these findings suggest that CD161^++^TCRvα7.2^+^ T cells in the mucosal surface of the LRT are poised to provide early control of infection and mediate development of subsequent optimal adaptive immune responses.

HIV infection leads to depletion of peripheral blood CD161^++^TCRvα^+^ T cells (34, 35), which is not reversed by anti-retroviral therapy (ART) (36). However, there are conflicting data on the impact of HIV on the functional capacity of CD161^++^TCRvα7.2^+^ T cells (37, 38). CD161^++^TCRvα7.2^+^ T cells obtained from untreated HIV-infected individuals were shown to retain their ability to produce IFN-γ and TNF upon stimulation with purified MR1 ligand (37). In contrast, following bacterial (*E. coli*) stimulation, CD161^++^TCRvα^+^ T cells from untreated HIV-infected individuals were shown to produce lower levels of IFN-γ, TNF, and IL-17 compared to healthy controls (38). Nevertheless, disruption of this important T cell subset likely contributes to increased susceptibility to infection in HIV-infected adults.

Despite important recent advances in CD161^++^TCRvα7.2^+^ T cell biology, the phenotype and functional characteristics of human airway CD161^++^TCRvα7.2^+^ T cells in health and asymptomatic HIV infection are not well-defined. Accumulating evidence suggests that CD161^++^TCRvα7.2^+^ T cells in mucosal sites may differ from those in circulation (12, 13, 39). To address this knowledge gap, we examined the frequency, phenotype and functional capacity of human airway CD161^++^TCRvα7.2^+^ T cells in healthy controls and asymptomatic HIV-infected adults before and 1 year after initiation of ART.

## Materials and Methods

### Study Participants

We recruited 80 individuals, classified as HIV-uninfected (*n* = 39), untreated asymptomatic HIV-infected (*n* = 41), and HIV-infected on ART (*n* = 6) at Queen Elizabeth Central Hospital, in Blantyre, Malawi. Participants were recruited from the hospital's Voluntary Counseling and Testing (VCT) clinic and they were all of black African origin. They were asymptomatic adults (≥18 years) with no clinical evidence of active disease, willing to undergo bronchoscopy and BAL for research purposes. Exclusion criteria for the study were current smoker, use of immunosuppressive drugs including ART at recruitment, and known or suspected pregnancy as screened by the study clinical team. Untreated HIV-infected individuals were commenced on ART in line with the “test and treat” strategy soon after undergoing bronchoscopy (within 36 h post HIV diagnosis). Participant demographics including age, sex, CD4 count, and plasma viral load are summarized in Table 1. All enrolled participants gave written informed consent as per protocol approved by College of Medicine Research Ethics Committee (COMREC; protocol P.03/16/1907) and Liverpool School of Tropical Medicine Research Ethics Committee (LSTM REC; protocol 15.054). Due to limitation in cell numbers, not all experiments were done on all samples. Specifically, the frequency of CD161^++^TCRvα7.2^+^ T cell data was generated on all 80 samples, the CD103 containing panel was used to generate data on a subset of 40 samples and the cytokine functional profile data was generated on a subset of 22 samples. Furthermore, for this study, we only had access to paired BAL and peripheral blood samples from HIV-uninfected individuals.

**Table 1:** Demographics of study participants.

	**HIV-uninfected controls (*n* = 39)**	**HIV-infected adults (*n* = 41)**	***p*-value**
Age (years)	32	32	0.4063^*^
Median (range)	(18–52)	(21–58)	
Sex (M:F)	30:9	21:20	0.0398^**^
CD4 count (cells/μl),	671	319	<0.0001^*^
Median (IQR)	(523–815)	(213–471)	
Plasma viral load (log 10	N/A	4.08	N/A
Copies/mL), Median (range)		(2.2–5.8)	

* *Unpaired T-test*.

** *Fishers exact test*.

*IQR, Interquartile range; ART, antiretroviral therapy; N/A, not applicable*.

### Sample Collection and Experimental Procedures

Bronchoscopy and bronchoalveolar lavage (BAL) were performed on all participants as previously described (5). Paired peripheral blood was also obtained from study participants for CD4 count and peripheral blood mononuclear cell (PBMC) isolation using density gradient centrifugation. Cell counts in BAL cells and PBMCs isolated from each sample were performed using a hemocytometer.

### Flow Cytometry

Immunophenotyping was performed as previously described (40). The antibodies used are described in Supplementary Table 1. BAL cells and PBMCs stimulations were performed using PMA/Ionomycin (Sigma Aldrich, UK) as a stimulant. Briefly, cells were incubated at a concentration of 1 × 10^6^ cells/200 μl in complete medium (200 μl per condition) in the presence of PMA/Ionomycin, BD GolgiPlug (BD Biosciences, UK) and BD GolgiStop (BD Biosciences, UK) for a total of 6 h at 37°C in a 5% CO_2_ incubator. After stimulation, cells were harvested and washed in PBS. Cells were labeled with the amine reactive dye LIVE/DEAD Fixable Aqua (Molecular Probes, Invitrogen, UK) prior to incubation with antibodies against surface proteins. Cytokines were stained after subsequent fixation/permeabilization with BD Cytofix/Cytoperm (BD Biosciences, UK).

### Cytometric Analyses

For all flow cytometric assays, at least 5,000 events in the CD8^+^ T cell gate were acquired using a LSRFortessa equipped with FACSDIVA software (BD Biosciences). Data were analyzed with FlowJo software (version 10.4.0, Tree Star). For cytokine functional profiles, PESTLE 1.7 and SPICE 5.3 (both NIAID, USA) were used for analysis. The programs PESTLE and SPICE were kindly provided by Mario Roederer, Vaccine Research Center, NIAID, NIH.

### Statistical Analysis

Statistical analyses and graphical presentation were performed using GraphPad Prism 5 (GraphPad Software, USA). Non-parametric tests were used to determine significance between groups using the Mann-Whitney two-tailed test (for two groups) or the Wilcoxon matched-pairs two-tailed test (for paired samples) (^*^p < 0.05; ^**^p < 0.01; ^***^p < 0.001). Pearson test was used to measured association between parameters.

## Results

### CD8^+^CD161^++^TCRvα7.2^+^ T Cells Are Present at Similar Frequencies in the Airway Lumen and Systemic Circulation

**Figure 1:**
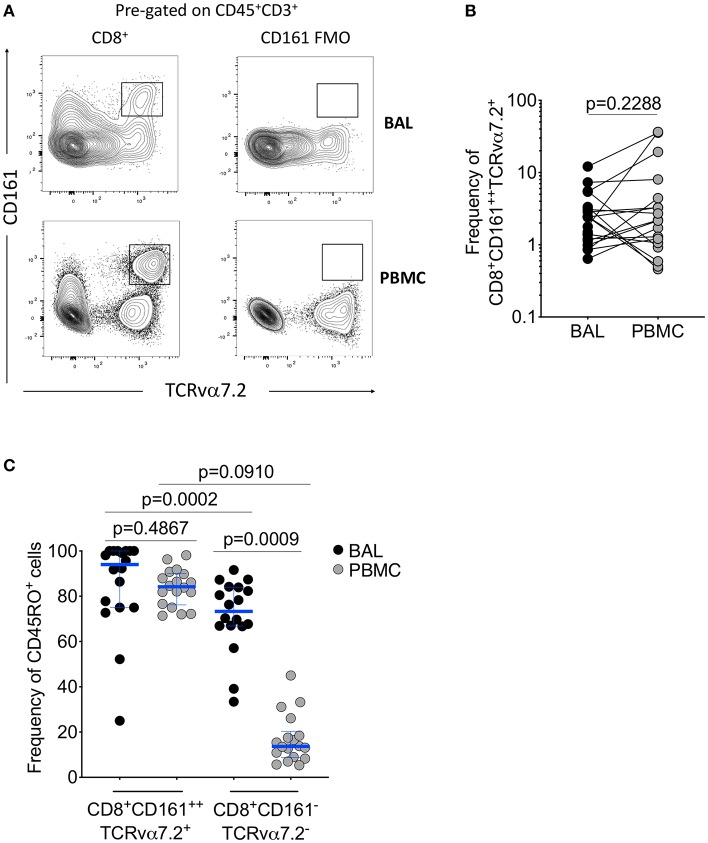
Identification of CD8^+^CD161^++^TCRvα7.2^+^ T cells in airway lumen and peripheral circulation. BAL cells and PBMCs from HIV-uninfected adults were stained with fluorochrome-conjugated antibodies against surface markers of interest. **(A)** Representative flow cytometry plots showing CD8^+^CD161^++^TCRvα7.2^+^ T cells in matched BAL and PBMC samples from a healthy HIV-uninfected adult. **(B)** Frequency of CD8^+^CD161^++^TCRvα7.2^+^ T cells in BAL compared to blood. **(C)** Memory phenotype of CD8^+^CD161^++^TCRvα7.2^+^ T cells in BAL compared to blood. Data were analyzed using Wilcoxon matched-pairs signed rank test (*n* = 20). BAL, bronchoalveolar lavage; PBMC, peripheral blood mononuclear cells.

To determine frequency of CD8^+^CD161^++^TCRvα7.2^+^ T cells from the airway lumen and systemic circulation, we obtained BAL fluid and peripheral blood from healthy HIV-uninfected adults, respectively (Table 1). Using flow cytometric analysis (Figure 1A; Supplementary Figure 1), we found similar frequencies of CD8^+^CD161^++^TCRvα7.2^+^ T cells in airway and peripheral blood samples (Figure 1B). As expected, the majority of CD8^+^CD161^++^TCRvα7.2^+^ T cells from both airways and blood exhibited a memory phenotype (CD45RO^+^) (94% [75–100] vs. 84% [76–90], *p* = 0.4867; Figure 1C). CD8^+^CD161^++^TCRvα7.2^+^ T cells were predominantly MR1 5-OP-RU positive (70%) and were the most abundant MR1 5-OP-RU positive population (Supplementary Figures 2, 3). However, due to low event numbers in the airway CD8^−^CD4^−^(DN) CD161^++^TCRvα7.2^+^ T cell population, we have only focused on the CD8^+^CD161^++^TCRvα7.2^+^ T cell population in this manuscript. These results confirm the presence of mature CD8^+^CD161^++^TCRvα7.2^+^ T cells in the airway of Malawian HIV-uninfected adults.

### CD103 Expressing CD8^+^CD161^++^TCRvα7.2^+^ T Cells Are the Predominantly Located in the Airway Lumen Compared to Systemic Circulation

**Figure 2:**
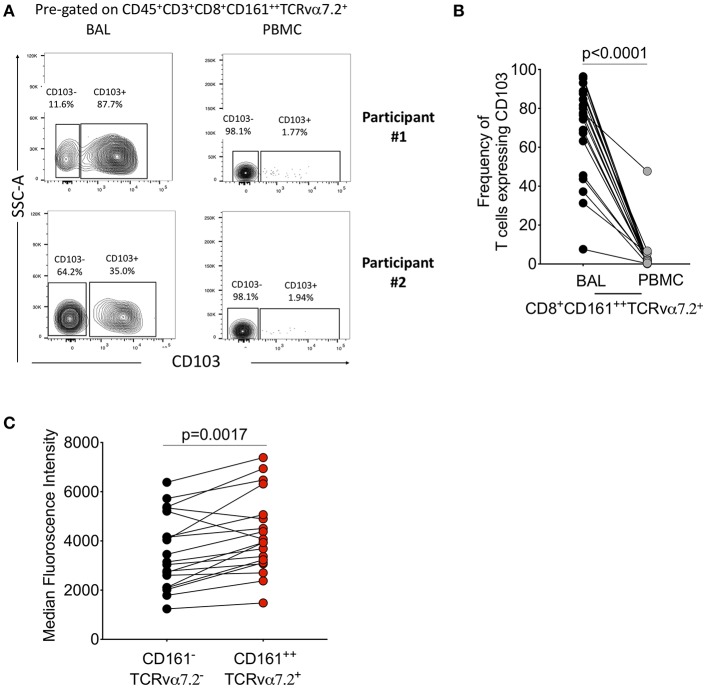
Characterization of CD8^+^CD161^++^TCRvα7.2^+^ T cells in airway lumen and peripheral circulation. BAL cells and PBMCs from HIV-uninfected adults were stained with fluorochrome-conjugated antibodies against surface markers of interest. **(A)** Representative flow cytometry plots showing CD103 expression in CD8^+^ T cells in matched BAL and PBMC samples from two healthy HIV-uninfected adults. **(B)** Proportion of CD103 expressing CD8^+^CD161^++^TCRvα7.2^+^ T cells in BAL and PBMCs. **(C)** CD103 expression intensity in CD8^+^CD161^++^TCRvα7.2^+^ T cells compared to classical CD8^+^ T cells from the airway lumen. Data were analyzed using Wilcoxon matched-pairs signed rank test (*n* = 19). BAL, bronchoalveolar lavage; PBMC, peripheral blood mononuclear cells.

We then further characterized the phenotype of the CD8^+^CD161^++^TCRvα7.2^+^ T cells by measuring CD103, a mucosal retention receptor commonly used to differentiate tissue-resident from infiltrating cells. CD103 is required for retention of cells in tissues as it binds to epithelial cell-expressed E-cadherin (41, 42). The proportion of CD8^+^CD161^++^TCRvα7.2^+^ T cells expressing CD103 was higher in airway compared to blood (77%[46–49] vs. 2% [0.32–2.6], *p* < 0.0001; Figures 2A,B). Furthermore, airway CD8^+^CD161^++^TCRvα7.2^+^ T cells expressed higher levels of CD103 compared to airway classical CD8^+^ T cells (Figure 2C). These results show that airway CD8^+^CD161^++^TCRvα7.2^+^ T cells are predominantly resident cells, but also contains a subset of potentially circulating/non-resident cells.

### CD103 Expressing Airway CD8^+^CD161^++^TCRvα7.2^+^ T Cells Possess a Distinct Cytokine Functional Profile

**Figure 3:**
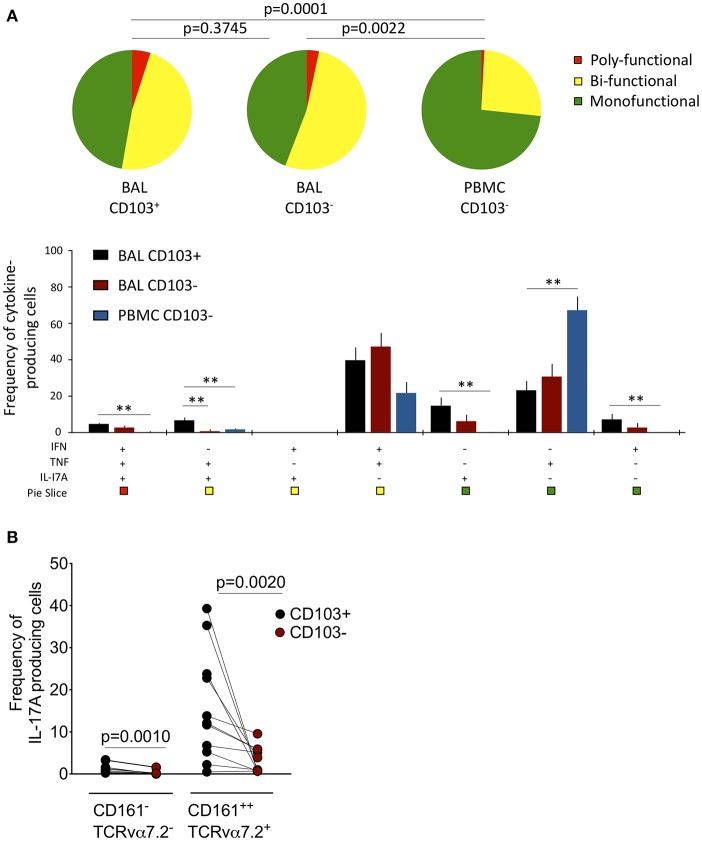
Functional profile of CD8^+^CD161^++^TCRvα7.2^+^ T cells in airway lumen and peripheral circulation. BAL cells and PBMCs from HIV-uninfected adults were stimulated with PMA/Ionomycin for 6 h and responses were measured by intracellular cytokine staining for TNF, IFN-γ, and IL-17A. The response was obtained by gating on singlets, lymphocytes, viable (LIVE/DEAD Aqua), CD3^+^ cells, CD8^+^ cells/ CD8^+^CD161^++^ TCRvα7.2^+^ T cells and combination of three cytokines. **(A)** *Each pie chart (top)* represents the mean distribution across subjects of mono-functional, bi-functional and poly-functional cytokine producing cells (color coded as shown) within the total response in a particular CD161^++^TCRvα7.2^+^ T cell population. *Bar charts (bottom)* represent the mean and standard error of the mean (SEM) of the contribution of the indicated subset (x-axis) toward the total response against the indicated CD161^++^TCRvα7.2^+^ T cell subsets (color coded as shown). Permutation test was performed among the pie charts and Wilcoxon test was done among the bar charts using SPICE software (^*^p < 0.05, ^**^p < 0.01). **(B)** Frequency of IL17A-producing cells in the CD103^+^ or CD103^−^ CD8^+^ T cell/CD8^+^CD161^++^TCRvα7.2^+^ T cell populations subtracting background responses obtained from the non-stimulated controls. The horizontal bars represent median, interquartile range and highest/lowest value. Data were analyzed using Mann Whitney test (*n* = 11). BAL, bronchoalveolar lavage; IFN, interferon-gamma; TNF, tumor necrosis factor; IL17, interleukin-17A.

Next, we tested whether CD103 expressing airway CD8^+^CD161^++^TCRvα7.2^+^ T cells were functionally distinct from CD8^+^CD161^++^TCRvα7.2^+^ T cells in peripheral blood (Supplementary Figure 4). The cytokine functional profile was different between airway and blood CD8^+^CD161^++^TCRvα^+^ T cells (Figure 3A). Specifically, a greater proportion of airway CD8^+^CD161^++^TCRvα7.2^+^ T cells were bi-functional compared to those from blood (BAL CD103^+^ 60% vs. Blood CD103^−^ 30%, *p* < 0.0001; BAL CD103^−^ 65% vs. Blood CD103^−^ 30%, *p* = 0.0018). Furthermore, the frequency of IFN-γ & TNF & IL-17A triple-producers, TNF & IL-17A duo-producers, IL-17A single-producers, and IFN-γ single-producers were higher in airway CD103^+^CD8^+^CD161^++^TCRvα7.2^+^ T cells than peripheral blood CD103^−^CD8^+^CD161^++^TCRvα7.2^+^ T cells (*p* < 0.01, *p* < 0.05, *p* < 0.01, and *p* < 0.05, respectively; Figure 3A). On the other hand, the frequency of TNF single-producers were higher in peripheral blood CD103^−^CD8^+^CD161^++^TCRvα7.2^+^ T cells than in CD103 expressing airway CD8^+^CD161^++^TCRvα7.2^+^ T cells (*p* < 0.01; Figure 3A).

We then investigated whether there was a functional difference between CD103 expressing CD8^+^CD161^++^TCRvα7.2^+^ T cells compared to non CD103 expressing cells from the airway lumen. The frequency of TNF and IL-17A duo-producers was higher in CD103 expressing than in non CD103 expressing cells (*p* < 0.01; Figure 3A). Furthermore, the frequency of CD8^+^CD161^++^TCRvα7.2^+^ T cells and classical CD8^+^ T cells from the airways producing IL-17A was higher in CD103 expressing compared to non CD103 expressing cells (Figure 3B), but this was not the case with IFN-γ or TNF (Supplementary Figure 5). Collectively, the results indicate that CD103 expressing airway CD8^+^CD161^++^TCRvα7.2^+^ T cells are functionally distinct, and that CD103 expression is associated with a propensity for IL-17A-production in airway CD8^+^ T cells.

### Selective Depletion of Airway CD103 Expressing CD8^+^CD161^++^TCRvα7.2^+^ T Cells in Untreated HIV-Infected Adults

**Figure 4:**
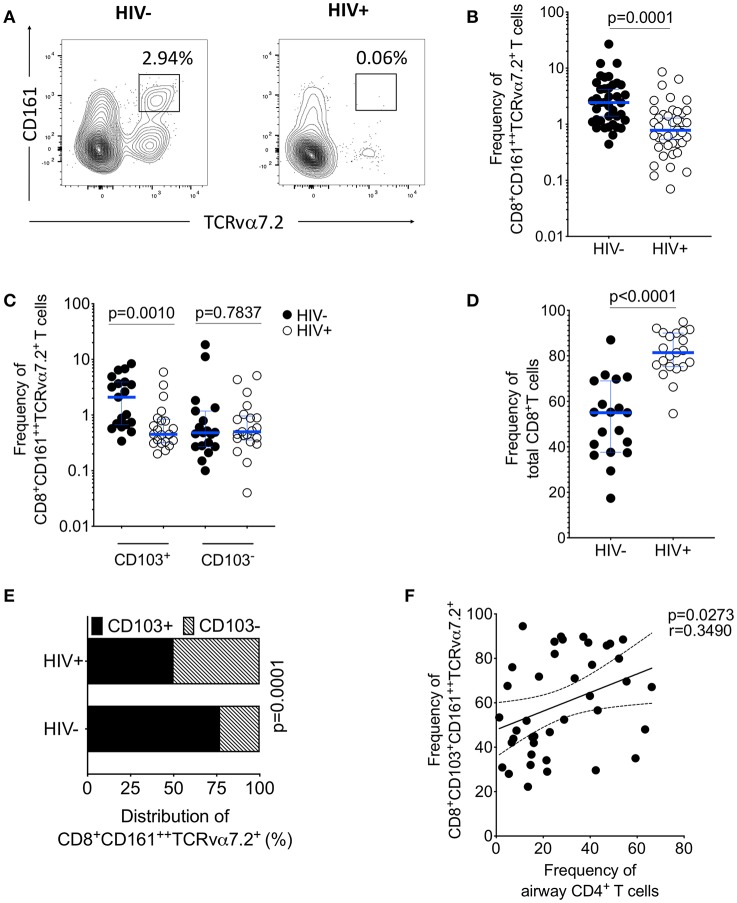
Depletion of airway CD103^+^CD8^+^CD161^++^TCRvα7.2^+^ T cells in untreated HIV-infected adults. BAL cells from HIV-uninfected and HIV-infected adults were stained with fluorochrome-conjugated antibodies against surface markers of interest. **(A)** Flow cytometry dot plots showing depletion of CD8^+^CD161^++^TCRvα7.2^+^ T cells. **(B)** Frequency of airway CD8^+^CD161^++^TCRvα7.2^+^ T cells in HIV-infected individuals compared to healthy controls (HIV^−^, *n* = 39; HIV^+^, *n* = 41). **(C)** Frequency of CD103^+/−^ airway CD8^+^CD161^++^TCRvα7.2^+^ T cells in HIV-infected individuals compared to healthy controls (HIV^−^, *n* = 19; HIV^+^, *n* = 21). **(D)** Frequency of total CD8^+^ T cells and CD4^+^ T cells in HIV-infected individuals compared to healthy controls (HIV^−^, *n* = 19; HIV^+^, *n* = 21). **(E)** Distribution of CD103^+/−^ airway CD161^++^TCRvα7.2^+^ T cells in HIV-infected individuals compared to healthy controls (HIV^−^, *n* = 19; HIV^+^, *n* = 21). **(F)** Association between frequency of airway CD103^+^ CD8^+^CD161^++^TCRvα7.2^+^ T cells and proportion of airway CD4^+^ T cells (*n* = 40; HIV^−^ *n* = 19, HIV^+^ *n* = 21). Data were analyzed using Mann Whitney test and the horizontal bars represent median, and interquartile range **(B,C,D)**. Data were analyzed using Fisher's exact test **(E)**. Data was analyzed using Pearson correlation test **(F)**. BAL, bronchoalveolar lavage.

HIV is associated with a depletion of peripheral blood CD161^++^TCRvα7.2^+^ T cells (34, 35), we investigated whether asymptomatic HIV infection alters the frequency of airway CD8^+^CD161^++^TCRvα7.2^+^ T cells. The frequency of airway CD8^+^CD161^++^TCRvα7.2^+^ T cells was significantly lower in untreated HIV-infected individuals compared to healthy controls (Figures 4A,B). In a subset of individuals, with paired CD8^+^ and DN CD161^++^TCRvα7.2^+^ T cells, we found no difference in this population between HIV-infected adults compared to healthy controls (Supplementary Figure 3). Specifically, the frequency of CD103 expressing airway CD8^+^CD161^++^TCRvα7.2^+^ T cells was lower in untreated HIV-infected adults than in HIV-uninfected controls, but the frequency of non CD103 expressing airway CD8^+^CD161^++^TCRvα7.2^+^ T cells was similar between the two groups (Figure 4C). In contrast, the frequency of total CD8^+^ T cell was higher in untreated HIV-infected adults than in HIV-uninfected controls (Figure 4D).

Furthermore, there was reduction in the distribution of CD103 expressing airway CD8^+^CD161^++^TCRvα7.2^+^ T cells in untreated HIV-infected adults compared to HIV-uninfected adults (52 vs. 78%, *p* < 0.001; Figure 4E). We also found that the frequency of CD103 expressing airway CD8^+^CD161^++^TCRvα7.2^+^ T cells was positively correlated with the frequency of airway CD4^+^ T cells (*p* = 0.0273, *r* = 0.3490; Figure 4F). Collectively, these findings show that depletion of airway CD8^+^CD161^++^TCRvα7.2^+^ T cells in untreated HIV-infected adults targets the CD103 expressing cells.

### HIV Viral Burden Is Associated With the Frequency of Airway CD8^+^CD161^++^TCRvα7.2^+^ T Cells

**Figure 5:**
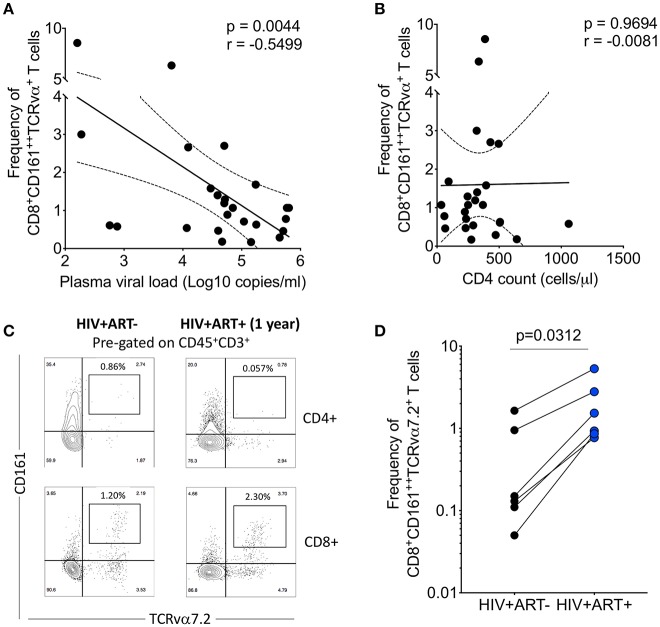
Depletion of airway CD8^+^CD161^++^TCRvα7.2^+^ T cells is inversely correlated with HIV plasma viral load. **(A)** Association between plasma viral load and frequency of airway CD8^+^CD161^++^TCRvα7.2^+^ T cells. Plasma viral load was log transformed (*n* = 25). **(B)** Association between peripheral blood CD4 count and frequency of airway CD8^+^CD161^++^TCRvα7.2^+^ T cells (*n* = 25). **(C)** Representative flow cytometry plots from an HIV-infected adult before and 1 year post ART initiation. **(D)** Frequency of airway CD8^+^CD161^++^TCRvα7.2^+^ T cells in from HIV-uninfected adults (before and 1 year post ART initiation) compared to healthy controls (HIV^+^ ART^−/+^, *n* = 6). Data was analyzed using Pearson correlation test **(A,B)**. Data were analyzed using Wilcoxon matched-pairs signed rank test for paired comparisons **(D)**. ART, anti-retroviral therapy.

HIV has direct pro-apoptotic impact on diverse cell types (43). We therefore determined whether the depletion of airway CD8^+^CD161^++^TCRvα7.2^+^ T cells is associated with HIV viral burden. We measured the association between HIV plasma viral load or peripheral blood CD4 count with frequency of airway CD8^+^CD161^++^TCRvα7.2^+^ T cells in 25 untreated HIV-infected adults with complete data. We found that HIV plasma viral load was inversely correlated with the frequency of airway CD8^+^CD161^++^TCRvα7.2^+^ T cells (Figure 5A). In contrast, the frequency of airway CD8^+^CD161^++^TCRvα7.2^+^ T cells was not directly correlated with peripheral blood CD4 count (Figure 5B).

To ascertain the impact of HIV viral burden on the depletion of airway CD8^+^CD161^++^TCRvα7.2^+^ T cells, we investigated whether suppressive antiretroviral therapy (ART) leads to recovery of airway CD8^+^CD161^++^TCRvα7.2^+^ T cells. We utilized 6 HIV-infected adults to which we had paired baseline CD8^+^CD161^++^TCRvα^+^ T cell data before commencement of ART and 1 year post ART initiation. All individuals had undetectable HIV plasma viral load at 1 year post ART initiation. Representative plots from an HIV-infected participant, before and 1 year following ART initiation (Figure 5C). Despite, having a small sample size, the frequency of airway CD8^+^CD161^++^TCRvα7.2^+^ T cells increased 1 year following initiation of ART (Figure 5D). Collectively, the findings show that HIV could directly or indirectly drive depletion of airway CD8^+^CD161^++^TCRvα7.2^+^ T cells and that ART leads to reconstitution of these cells.

### The Cytokine-Functional Profile of Airway CD8^+^CD161^++^TCRvα7.2^+^ T Cells Is Minimally Impacted by HIV Infection

**Figure 6:**
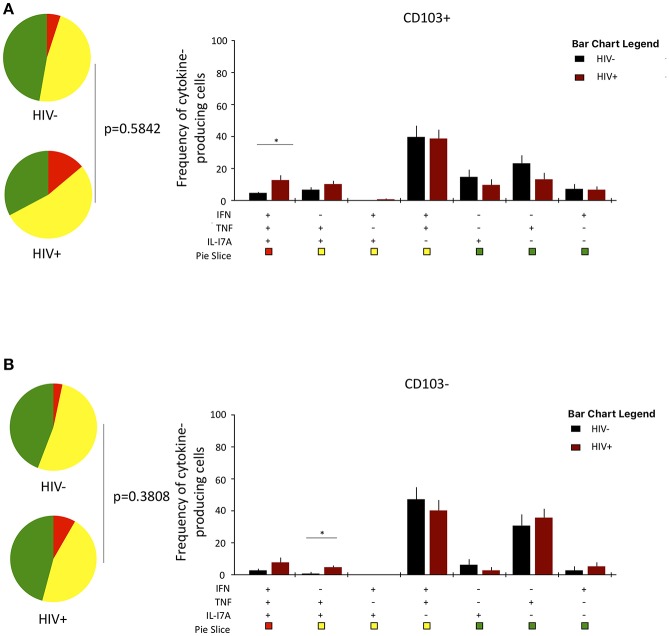
Function profile of CD8^+^CD161^++^TCRvα7.2^+^ T cells in healthy HIV-uninfected individuals compared to HIV-infected adults. BAL cells were stimulated with PMA/Ionomycin for 6 h and responses were measured by intracellular cytokine staining for IL-17A, IFN-γ, and TNF. The phenotype of the responding cells was obtained by gating on singlets, lymphocytes, viable (LIVE/ DEAD Aqua), CD3^+^ cells, CD8^+^ cells, IL-17A^+^, and then a combination of CD161 and CD103. **(A)** CD103^+^ and **(B)** CD103^−^. *Each pie chart (top)* represents the mean distribution across subjects of mono-functional, bi-functional and poly-functional cytokine producing cells (color coded as shown) within the total response in a particular CD8^+^CD161^++^TCRvα7.2^+^ T cell subset. *Bar charts (bottom)* represent the mean and standard error of the mean (SEM) of the contribution of the indicated subset (x-axis) toward the total response against the indicated CD8^+^CD161^++^TCRvα7.2^+^ T cell subsets (color coded as shown) (*n* = 11). Permutation test was performed among the pie charts and Wilcoxon test was done among the bar charts using SPICE software (^*^p < 0.05). BAL, bronchoalveolar; IFN, interferon-gamma; TNF, tumor necrosis factor; IL17, interleukin-17A.

Lastly, we investigated whether HIV infection alters cytokine functional profile of airway CD8^+^CD161^++^TCRvα7.2^+^ T cells. Overall, the cytokine-producing functional profile in airway CD8^+^CD161^++^TCRvα7.2^+^ T cells was not different between untreated HIV-infected adults compared to HIV-uninfected individuals, in both the CD103^+^ and CD103^−^ cells (Figures 6A,B). However, frequency of CD103^+^IFN-γ^+^TNF^+^IL17A^+^-poly-functional and CD103^−^IFN-γ^−^TNF^+^IL17A^+^-bi-functional airway CD8^+^CD161^++^TCRvα7.2^+^ T cells was higher in HIV-infected adults than HIV-uninfected controls (Figures 6A,B). These results show that HIV infection, minimally but differentially, impacts cytokine-functional profile of airway CD8^+^CD161^++^TCRvα7.2^+^ T cell subsets.

## Discussion

CD8^+^CD161^++^TCRvα7.2^+^ T cells are part of the innate-like T cell family with important functional relevance in defense against a diverse repertoire of pathogens. There is limited data on the phenotypic and functional characteristics of human airway CD8^+^CD161^++^TCRvα7.2^+^ T cells and how HIV impacts these cells in asymptomatic individuals from high respiratory disease-burdened settings. This study sheds new light on the functional capacity of human airway CD8^+^CD161^++^TCRvα7.2^+^ T cells, their compartmentalized nature, and demonstrates selective depletion of CD103 expressing airway CD8^+^CD161^++^TCRvα7.2^+^ T cells in untreated HIV-infected African adults, and reconstitution of airway CD8^+^CD161^++^TCRvα7.2^+^ T cells 1 year following ART initiation.

Consistent with observations that CD8^+^CD161^++^TCRvα7.2^+^ T cells possess different functional characteristics related to their site of origin (12, 13, 39), we show that airway CD8^+^CD161^++^TCRvα7.2^+^ T cells are phenotypically and functionally different from those in systemic circulation. Specifically, the majority of the airway CD8^+^CD161^++^TCRvα7.2^+^ T cells express mucosal retention receptor CD103, and are more poly-functional than those from blood. Furthermore, CD103 expressing CD8^+^CD161^++^TCRvα7.2^+^ T cells exhibited propensity for IL-17A production. This is constituent with recent observations from the oral mucosa, showing MAIT cells that exhibit a tissue-resident-activated profile biased toward IL-17 production (44). IL-17 plays an important role in mucosal defense as it acts as a bridge between innate and adaptive immunity (45–47) and also induces production of antimicrobial peptides (48). Due to the innate-like function of airway CD8^+^CD161^++^TCRvα7.2^+^ T cells, production of IL-17A and expression of CD103 likely confers these cells readiness and immediate availability to respond quickly to pathogens at a major portal of entry. This is evidenced by observations in animal models that show poor early control of respiratory infections in CD161^++^TCRvα7.2^+^ T cell-deficient animals compared to wild type controls (32, 33).

Certainly, disruption of airway CD8^+^CD161^++^TCRvα7.2^+^ T cell homeostasis could contribute to increased susceptibility to respiratory infections. Consistent with data from non-human primates (49), we show a depletion in airway CD8^+^CD161^++^TCRvα7.2^+^ T cells in untreated HIV-infected adults. Specifically, we show that CD103 expressing airway CD8^+^CD161^++^TCRvα7.2^+^ T cells are selectively depleted in untreated HIV-infected adults. We also observed an inverse correlation between HIV plasma viral load and frequency of airway CD8^+^CD161^++^TCRvα7.2^+^ T cells, supporting a direct or indirect role of HIV in depletion of these cells. CD103 is required for retention of cells in tissues as it binds to epithelial cell-expressed E-cadherin (41, 42). CD4^+^ T cells are important for the formation of functional CD103^+^CD8^+^ T cells, and we have shown in this study that the frequency of CD103 expressing airway CD8^+^CD161^++^TCRvα7.2^+^ T cells was positively correlated with the frequency of airway CD4^+^ T cells. It has been shown that absence of CD4^+^ T cells leads to reduced expression of CD103 on CD8^+^ T cells and subsequent mislocalization of these cells away from airway epithelia (50). Downregulation of CD103 could potentially result in egress of CD8^+^CD161^++^TCRvα7.2^+^ T cells from the airway lumen. It is also plausible that HIV-induced immune activation could lead activation-induced cell death (AICD) (51), immune exhaustion (31) or to upregulation of intergrins in the lung tissue, resulting in impaired trafficking of CD8^+^ MAIT cells from the tissue into the airway lumen. This could result in poor replenishment of CD103 expressing airway CD8^+^CD161^++^TCRvα7.2^+^ T cells in the airway lumen.

Consistent with a potential direct or indirect role of HIV on alteration of airway CD8^+^CD161^++^TCRvα7.2^+^ T cell homeostasis, suppressive ART was associated with a reconstitution of airway CD8^+^CD161^++^TCRvα7.2^+^ T cells. CD161^++^TCRvα7.2^+^ T cell reconstitution has also been observed in colonic tissue from HIV-infected individuals on ART (52). In contrast to peripheral blood, where CD161^++^TCRvα^+^ T cell are not reconstituted by ART (36, 52), both colon and respiratory mucosa have diverse microbiota rich with potential CD161^++^TCRvα7.2^+^ T cell stimulating commensal microbes (53). Microbial exposure shapes and maintains the CD161^++^TCRvα7.2^+^ T cell repertoire (22). Germ-free mice lack CD161^++^TCRvα7.2^+^ T cell, but acquire them after reconstitution with commensal bacterial strains (20, 54). HIV infection disrupts the lung microbiota (55) and this could compromise homeostatic maintenance of airway CD161^++^TCRvα7.2^+^ T cells. ART-mediated suppression of viremia could lead to changes in the respiratory microbiota that favor recovery of CD161^++^TCRvα7.2^+^ T cell populations in the mucosal compartments.

Interestingly, HIV was not associated with impairment of cytokine-secreting functional potential in airway CD8^+^CD161^++^TCRvα7.2^+^ T cells. This is consistent with data showing retention of IFN-γ and TNF-producing function in peripheral blood-derived CD161^++^TCRvα7.2^+^ T cells stimulated with purified MR1 ligand in untreated HIV-infected individuals (37). However, it is inconsistent with results obtained following bacterial (*E. coli*) stimulation of peripheral blood-derived CD161^++^TCRvα7.2^+^ T cells, which showed lower levels of IFN-γ, TNF and IL-17 in untreated HIV-infected individuals compared to healthy controls (38). It is therefore, plausible, that the overpowering nature of PMA/Ionomycin stimulation could mask the small differences in function between airway CD8^+^CD161^++^TCRvα7.2^+^ T cells from HIV-infected adults and HIV-uninfected controls. On the other hand, PMA/Ionomycin stimulation brings out the full cytokine-secreting functional potential of the airway CD8^+^CD161^++^TCRvα7.2^+^ T cells and by-passes the need for functional antigen-presentation, which could potentially skew results in HIV-infected adults.

While our study provides useful insights into the phenotype and function of airway CD8^+^CD161^++^TCRvα7.2^+^ T cells, we acknowledge some limitations. First, we did not use MR1 tetramers or other surface markers (such as CD26) to identify MAIT cells. However, we have used CD161 and TCRvα7.2, classical MAIT cell markers used by most studies in literature, as well as provided a representative flow cytometry plot showing that over 70% of the CD8^+^CD161^++^TCRvα7.2^+^ T cells stain positive for MR1-5-OP-RU Tetramer (Supplementary Figure 2). Second, we were only able to perform serial bronchoscopy in a subset of individuals due to challenges associated with serial research bronchoscopy. However, the data from the small subset of participants was consistent with the earlier observations that showed an inverse correlation between HIV plasma viral load and frequency of airway CD8^+^CD161^++^TCRvα7.2^+^ T cells. Third, we were not able to further characterize the airway DN CD161^++^TCRvα7.2^+^ T cells due to the limitation in event numbers. However, in a subset, we were able to show that HIV infection did not impact this airway DN CD161^++^TCRvα7.2^+^ T cell population (Supplementary Figure 3).

In conclusion, we have shown that CD103 expressing airway CD8^+^CD161^++^TCRvα7.2^+^ T cells are functionally distinct from those in systemic circulation, and are preferentially depleted during untreated asymptomatic HIV infection. Disruption of airway CD8^+^CD161^++^TCRvα7.2^+^ T cell homeostasis likely creates a conducive environment for susceptible respiratory pathogens, and could partly contribute to the increased propensity for LRTIs in HIV-infected adults.

## Data Availability

The datasets generated for this study are available on request to the corresponding author.

## Author Contributions

KJ, HM, LM, and AM: conception and design. KJ, HM, LM, AM, AH, JP, RK, DM, EC, AK, and RM: analysis and interpretation. KJ, LM, and HM: drafting the manuscript for important intellectual content. KJ, HM, LM, AM, RK, JP, DM, AH, EC, RM, and AK: final approval.

### Conflict of Interest Statement

The authors declare that the research was conducted in the absence of any commercial or financial relationships that could be construed as a potential conflict of interest.
