# Supplementary material for: Airway CD8+CD161++TCRvα7.2+ T Cell Depletion During Untreated HIV Infection Targets CD103 Expressing Cells
Source: Front Immunol. 2019 Aug 21;10:2003. doi: 10.3389/fimmu.2019.02003 (PMC6713019; doi:10.3389/fimmu.2019.02003)

Supplementary material

Supplementary Figure 1. Representative flow cytometry dot plots showing gating strategy for identifying $CD8^+CD161^{++}$ $TCRv\alpha7.2^+$ T cells from an HIV-uninfected healthy control. PBMCs were stained with fluorochrome-conjugated antibodies against surface markers of interest. The flow cytometry dot plots were obtained by gating on singlets, $CD45^+$ cells, lymphocytes, $CD3^+$ cells, $CD8^+$ cells or $CD4^+$ cells, then $CD161^{++}$ $TCRv\alpha7.2^+$, followed by CD45RO.

Supplementary Figure 2. Representative flow cytometry dot plots showing MR1 5-OP-RU tetramer staining of airway $CD161^{++}$ $TCRv\alpha7.2^+$ T cells in an HIV-uninfected control. Frozen BAL cells were stained with fluorochrome-conjugated antibodies against surface markers of interest, together with MR1 5-OP-RU tetramer or MR1 6FP tetramer control. The flow cytometry dot plots were obtained by gating on singlets, lymphocytes, live $CD3^+$ cells, $CD4^-CD8^-$ or $CD8^+$ cells or $CD4^+$ cells, followed by $CD161^{++}$ $TCRv\alpha7.2^+$. **A)** Flow plots showing MR1 5-OP-RU tetramer and MR1 6FP tetramer staining control. The plots show that the majority of the airway $CD8^+CD161^{++}$ $TCRv\alpha7.2^+$ T cells MR1 5-OP-RU tetramer positive cells are also CD8 positive. **B)** Flow plots showing MR1 5-OP-RU tetramer staining among subsets of airway $CD161^{++}$ $TCRv\alpha7.2^+$ T cells, $CD8^+CD4^-$, $CD8^-CD4^-$ and $CD8^-CD4^+$

Supplementary Figure 3. Frequency and proportion of $CD8^+$ and DN airway $CD161^{++}$ $TCRv\alpha7.2^+$ T cells. A) Proportion of airway $CD8^+$ and DN $CD161^{++}TCRv\alpha7.2^+$ T cells in HIV-infected individuals and healthy controls **B)** Frequency of airway $CD8^+$ and DN $CD161^{++}TCRv\alpha7.2^+$ T cells in HIV-infected individuals and healthy controls. Data were analysed using Wilcoxon matched-pairs signed rank test for paired comparisons and Mann Whitney test for the unpaired comparison, the horizontal bars represent median and interquartile range. (HIV-, n=17; HIV+, n=16). DN, Double Negative, $CD8^-CD4^-$

Supplementary Figure 4. Representative flow cytometry dot plots showing gating strategy for identifying cytokine-secreting airway and peripheral blood $CD8^+CD161^{++}$ $TCRv\alpha7.2^+$ T cells from an HIV-uninfected healthy control. BAL cells were stimulated with PMA/Ionomycin for 6 hours and responses were measured by intracellular cytokine staining for IL-17A, IFN-γ and TNF. The response was obtained by gating on singlets, lymphocytes, viable (LIVE/ DEAD Aqua) $CD45^+$ cells, $CD3^+$ cells, $CD8^+$ cells or $CD4^+$ T cells, then $CD103^{+/-}CD161^{++}$ $TCRv\alpha7.2^+$ cells and each single cytokine.

Supplementary Figure 5. IFN-γ and TNF-producing airway T cells in $CD103^+$ versus $CD103^-$ cells. BAL cells were stimulated with PMA/Ionomycin for 6 hours and responses were measured by intracellular cytokine staining. **A)** Each data point represents the frequency of TNF-producing cells within each subset (n=11). **A)** Each data point represents the frequency of IFN-γ-producing cells within each subset (n=11).

Supplementary Table 1: Fluorochromes

Marker	Fluorochrome	Clone	Provider	Filter	Isotype
CD3	PerCP/Cy5.5	HIT3a	Biolegend	695/40	Mouse IgG2a, κ
CD4	Brilliant Violet 421	RPA-T4	Biolegend	450/50	Mouse IgG1, κ
CD8	APC/Cy7	HIT8a	Biolegend	780/60	Mouse IgG1, κ
CD161	PE/Dazzle 594	HP-3G10	Biolegend	610/20	Mouse IgG1, κ
TCRvα7.2	Brilliant Violet 605	3C10	Biolegend	605/20	Mouse IgG1, κ
IL-17A	PE	BL168	Biolegend	575/26	Mouse IgG1, κ
TNF	Alexa Flour 488	MAb11	BD Biosciences	530/30	Mouse IgG_1, κ
IFN-γ	FITC	B27	BD Biosciences	530/30	Mouse IgG1, κ
CD103	PE/Cy7	Ber-ACT8	Biolegend	780/60	Mouse IgG1, κ
CD45	Alexa Flour 700	2D1	Biolegend	730/45	Mouse IgG1, κ
CD45RO	FITC	UCHL1	BD Biosciences	530/30	Mouse IgG2a, κ
TCR Vα7.2	PE	3C10	Biolegend	575/26	Mouse IgG1, κ
CD4	Alexa Flour 700	RPA-T4	Biolegend	730/45	Mouse IgG1, κ
Live & Dead	BV510	NA	Invitrogen	510/50	NA

Supplementary Figure 1

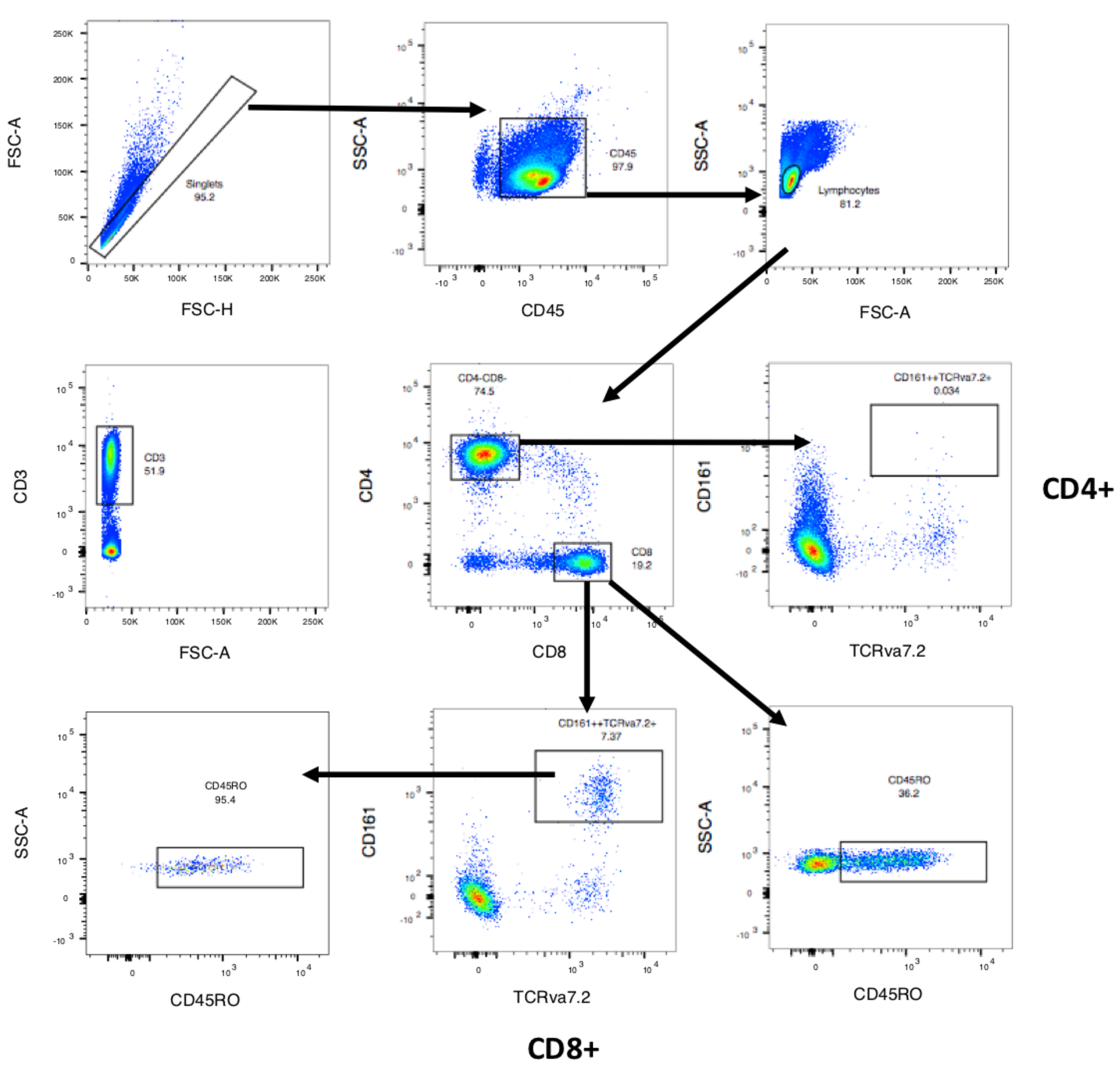

Supplementary Figure 2

A.

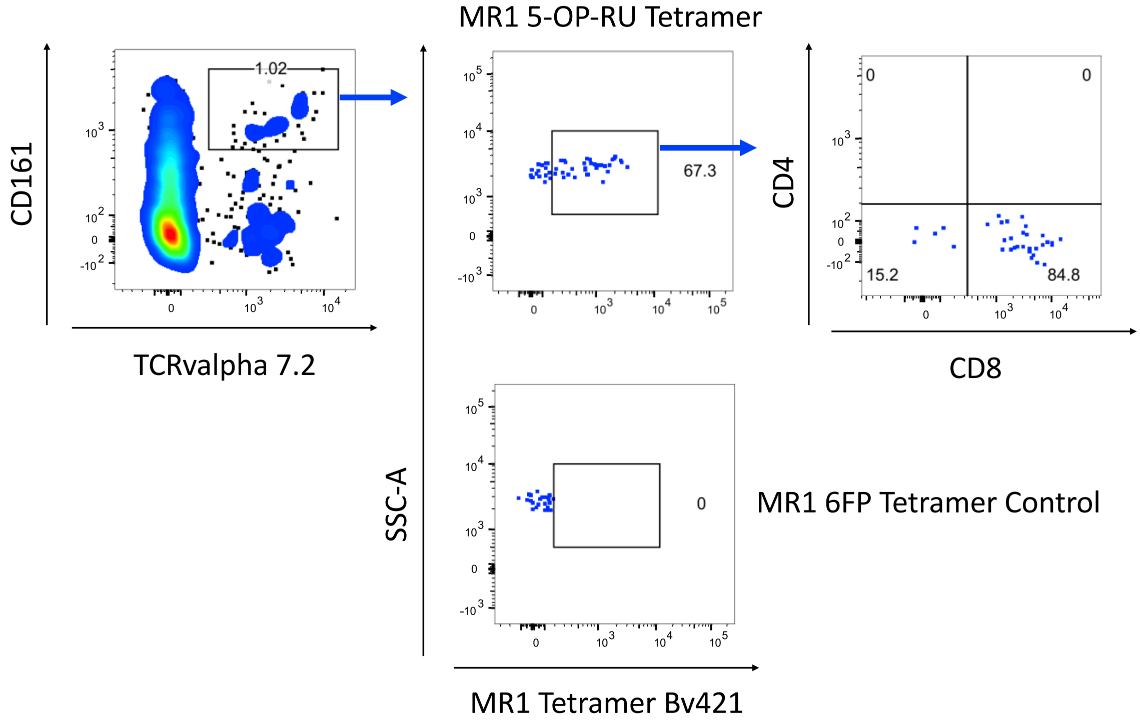

B.

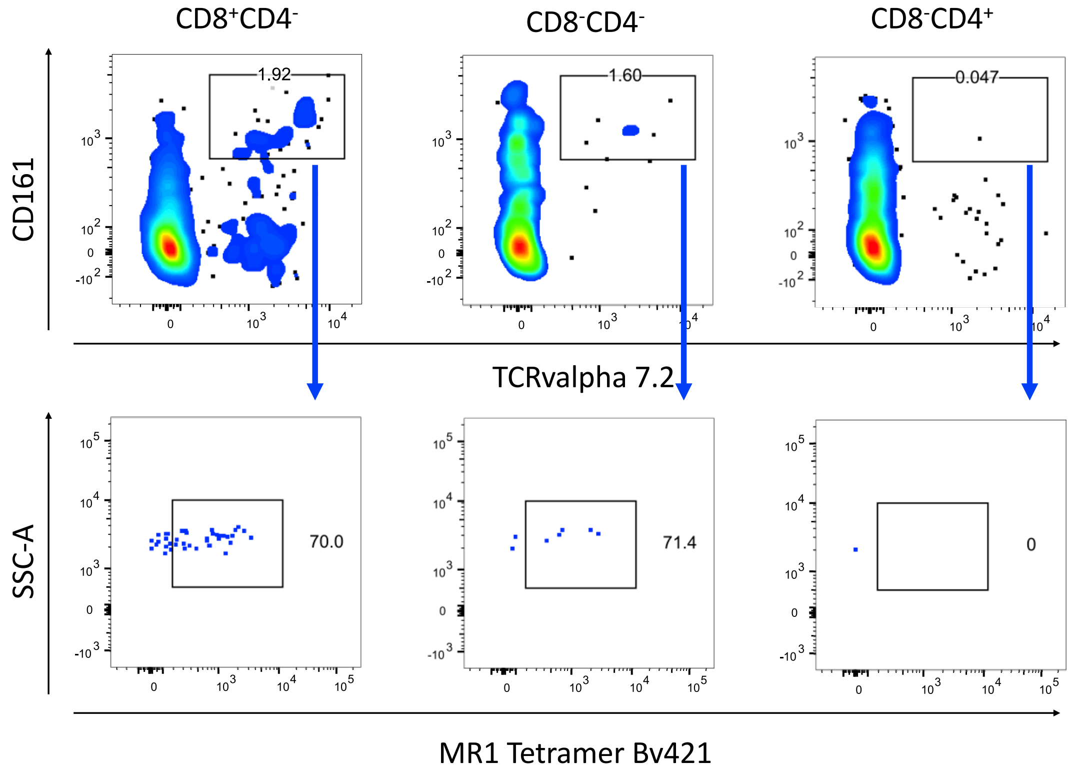

Supplementary Figure 3

A.

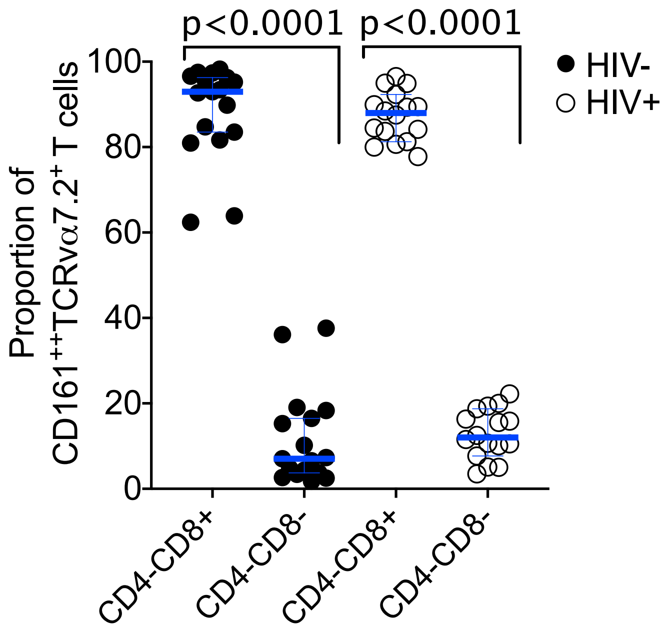

B.

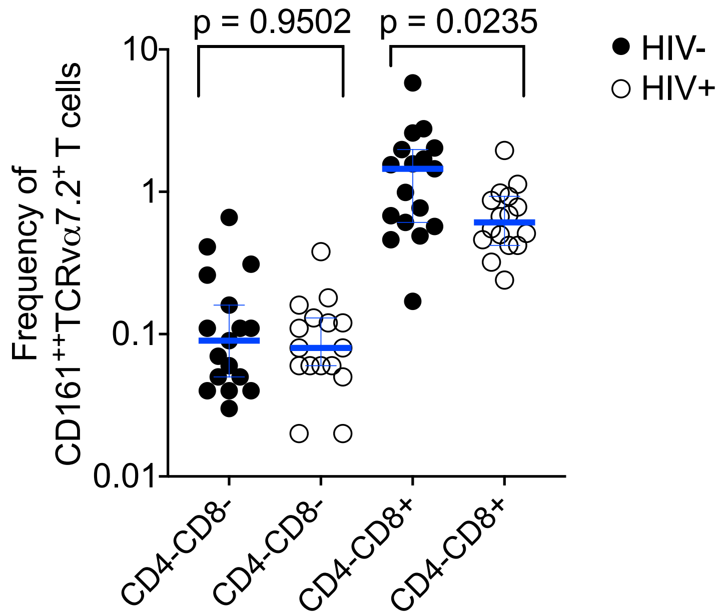

Supplementary Figure 4

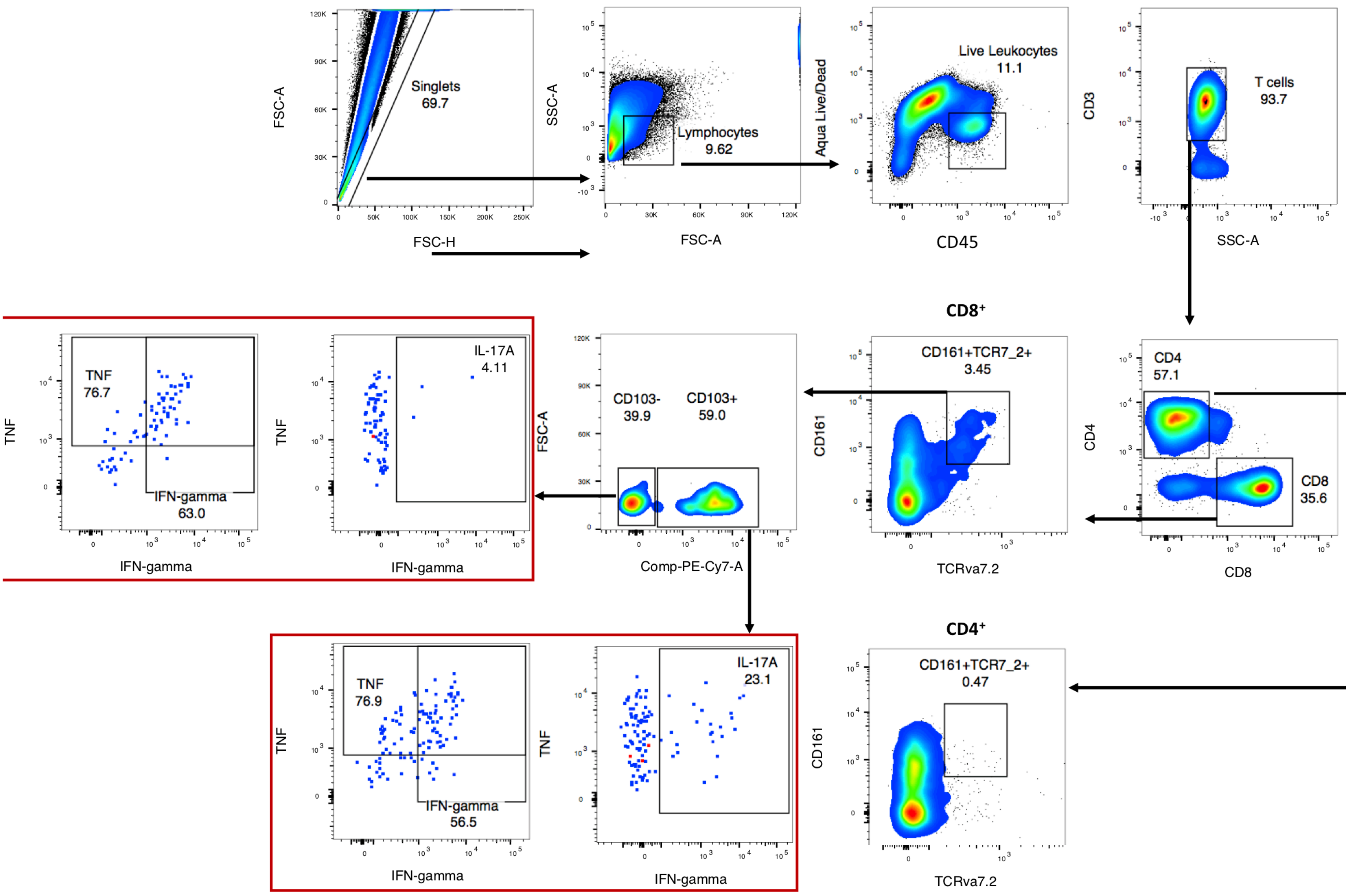

Supplementary Figure 5

A.

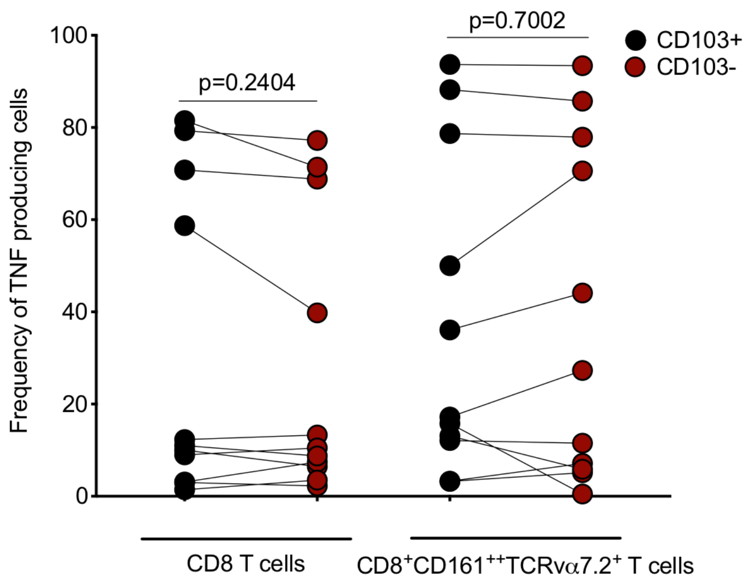

B.

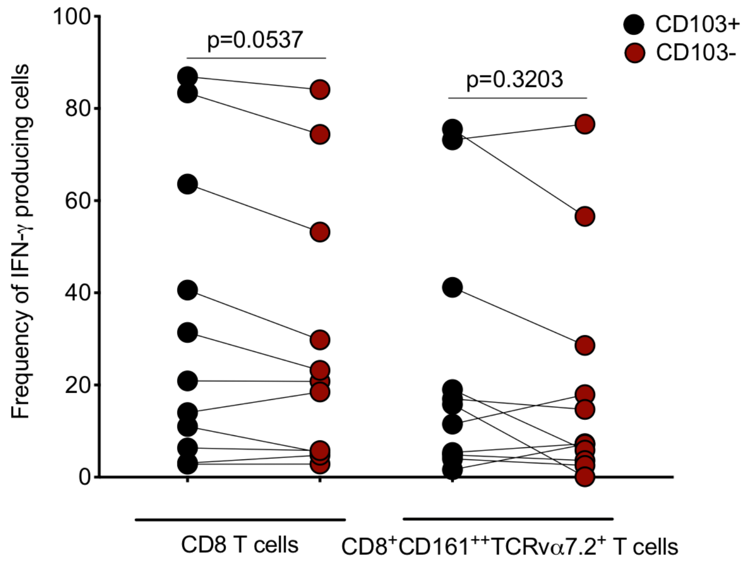

Supplement: Supplementary file 1 [file Presentation_1.pdf]
